# Cross-Modal Calibration of Vestibular Afference for Human Balance

**DOI:** 10.1371/journal.pone.0124532

**Published:** 2015-04-20

**Authors:** Martin E Héroux, Tammy C. Y. Law, Richard C. Fitzpatrick, Jean-Sébastien Blouin

**Affiliations:** 1 School of Kinesiology, University of British Columbia, Vancouver, Canada; 2 Neuroscience Research Australia and School of Medical Sciences, University of New South Wales, Sydney, Australia; 3 Djavad Mowafaghian Centre for Brain Health & Institute for Computing, Information and Cognitive Systems, University of British Columbia, Vancouver, Canada; Tokai University, JAPAN

## Abstract

To determine how the vestibular sense controls balance, we used instantaneous head angular velocity to drive a galvanic vestibular stimulus so that afference would signal that head movement was faster or slower than actual. In effect, this changed vestibular afferent gain. This increased sway 4-fold when subjects (N = 8) stood without vision. However, after a 240 s conditioning period with stable balance achieved through reliable visual or somatosensory cues, sway returned to normal. An equivalent galvanic stimulus unrelated to sway (not driven by head motion) was equally destabilising but in this situation the conditioning period of stable balance did not reduce sway. Reflex muscle responses evoked by an independent, higher bandwidth vestibular stimulus were initially reduced in amplitude by the galvanic stimulus but returned to normal levels after the conditioning period, contrary to predictions that they would decrease after adaptation to increased sensory gain and increase after adaptation to decreased sensory gain. We conclude that an erroneous vestibular signal of head motion during standing has profound effects on balance control. If it is unrelated to current head motion, the CNS has no immediate mechanism of ignoring the vestibular signal to reduce its influence on destabilising balance. This result is inconsistent with sensory reweighting based on disturbances. The increase in sway with increased sensory gain is also inconsistent with a simple feedback model of vestibular reflex action. Thus, we propose that recalibration of a forward sensory model best explains the reinterpretation of an altered reafferent signal of head motion during stable balance.

## Introduction

Human balance control is based on the combined inflow of different sensory systems. Proprioceptive, visual, haptic, and vestibular sensors are considered the most important sources of information [1]. In many situations they provide largely redundant information so that loss of one is not critical. In situations where one sensory channel becomes critical for balance or another become false or unreliable, the CNS might selectively attend or ignore specific channels through a process of “reweighting” [2–6]. Current concepts of motor control are based on forward sensory and motor models [7–8] whereby the CNS responds to the sensed difference between the predicted sensory inflow for an action and the actual sensed inflow. It is not clear how the brain implements the forward sensory model and, particularly in the example of multisensory balance control, how different sensory channels contribute to the forward sensory expectation. Human standing with its multisensory control of a clearly defined motor task is an ideal model to explore these questions.

In the semicircular canals, the spontaneous discharge of primary afferent neurones is modulated bi-directionally by head angular motion. Angular acceleration of the head is transduced through a “mechanical integration” by inertial and visco-elastic properties of the cupula-endolymph system to generate a primary afferent signal that approximately represents the angular velocity of head motion [9–10]. How does the CNS use this signal to activate the postural muscles for balance control? Being a rate-coded signal, it is worth asking how the CNS knows what motion corresponds with what rate to generate motor output appropriate for balance control.

Small mastoidal electrical currents distort vestibular afferent signals without affecting other sensory systems [11]. When applied as a binaural bipolar stimulus it evokes a pattern of afferent firing that signals natural head rotation in roll [12–13]. When the CNS is repeatedly exposed to daily or weekly sessions of pseudorandom or sinusoidal vestibular stimulation, the stimulation initially causes severe postural disturbances, but this effect dissipates and balance returns to baseline levels after 7–8 sessions [2–3]. These results appear to reflect a reweighting of sensory inputs [2–3]. Because vestibulo-ocular reflexes remain affected despite repeated exposure to these same artificial vestibular stimuli, reweighting likely involves slow cerebellar processes rather than actual changes at the level of the vestibular end-organs or reflexes [2]. The purpose of the present experiment was to further explore balance control and how the CNS adapts to altered vestibular signals. To achieve this objective, we exposed standing subjects to a galvanic vestibular stimulus that was coupled to head motion so that vestibular afference signalled body sway that was slower or faster than actual. As galvanic stimulation bypasses mechanotransduction [14–15], measured head movement was passed through the canal mechanotransduction transfer function [9] to evoke an afferent firing pattern appropriate for the movement. We hypothesised that changing the size of the afferent response to head motion will result in unstable balance. To discover how the brain deals with such altered afference, we then provided a conditioning period with a reliable visual or somatosensory signal of body movement while the false vestibular signal continued. After this conditioning, we expected balance to be stable without the visual or somatosensory cues despite the continuing false vestibular signal and to become unstable again when the galvanic stimulus was removed. Our results confirmed these hypotheses, suggesting that, during the brief conditioning, the vestibular signal has been recalibrated to align it with other sensory channels and the sensation of stable balance.

## Methods

### Subjects

Eight adults (21–36 years, 2 female) with no history of neurological disease or injury participated in the first experiment and five of them participated in the second. The experiments were approved by the University of British Columbia Clinical Research Ethics Board and were conducted in accord with the Declaration of Helsinki after obtaining informed written consent.

### Changing vestibular gain

We used galvanic vestibular stimulation (GVS) to amplify or attenuate the vestibular afferent response to the head rotation that occurs with body sway during standing. In binaural bipolar configuration, the altered discharge of canal afferents evokes a net signal of head rotation about a specific axis [11]. This GVS axis is directed posteriorly and inclined ~18° above Reid's plane; approximately the axis of head roll [13, 16]. While GVS also stimulates otolith afferents [17], the dominant postural responses are to this head roll while linear responses of otolithic origin are relatively minor [18–21]. Based on this evidence, we measured head rotation (angular velocity) about the GVS axis in standing subjects and used it to deliver a real-time galvanic stimulus to increase or decrease the normal canal response to that head rotation.

**Fig 1:**
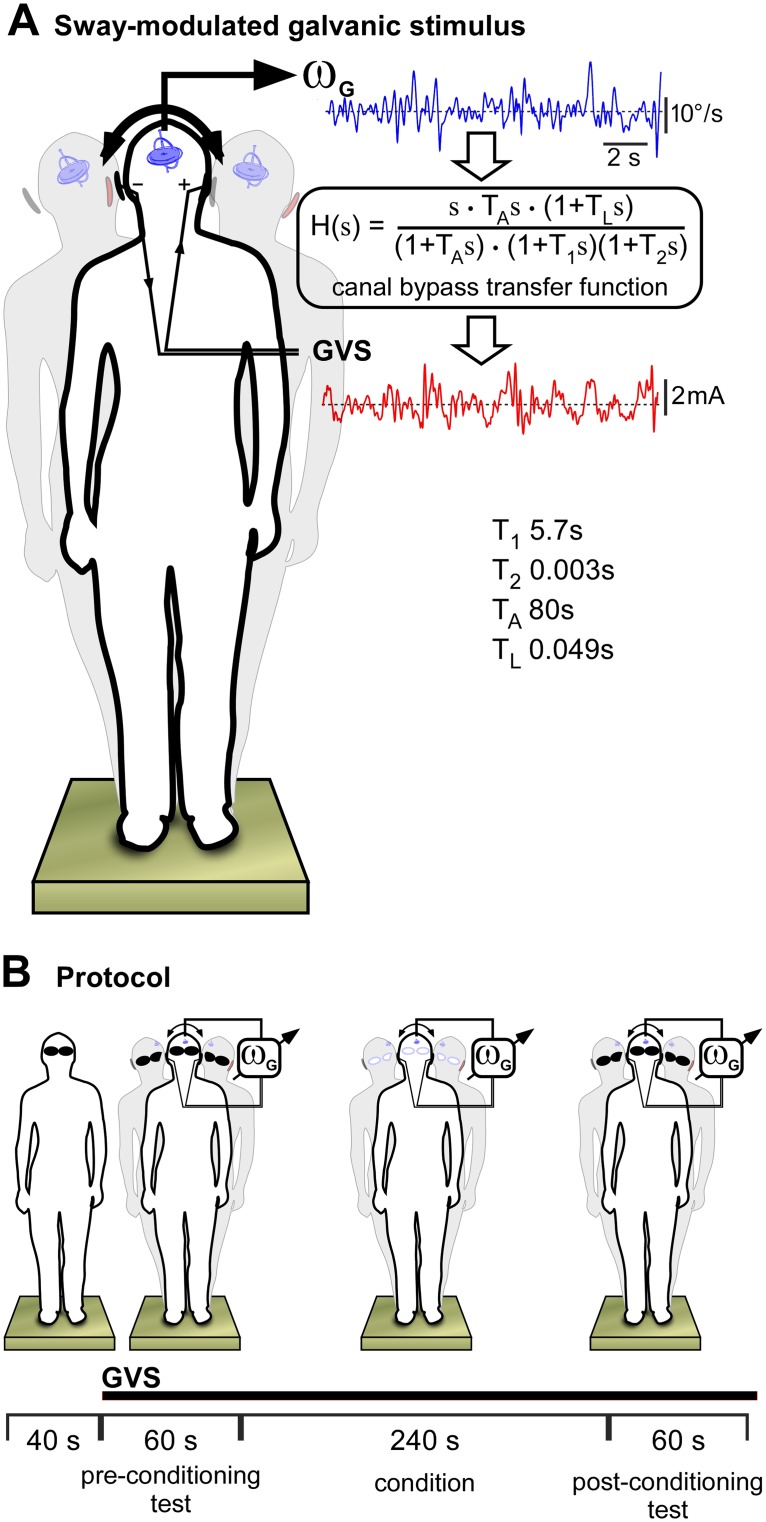
Experiment. (A) Subjects stood on a foam pad. Signals from a tri-axial angular velocity sensor secured to the head were used to determine instantaneous head angular velocity (ω_G_) about the GVS axis. This signal was passed through the canal transfer function identified by Goldberg and Fernandez [10] to create the galvanic stimulus (GVS) that would evoke the pattern of afferent neuron firing that would arise from the head motion, and then scaled to 0.125 mA per deg.s-1. The bipolar stimulus (GVS) is delivered at the mastoid processes. This galvanic response, added to the natural stimulus, amplified the afferent response to the natural movement, and when subtracted (reverse stimulus polarity) attenuated the afferent response. (B) In each trial, subjects stood for 40 s as baseline before the GVS was delivered. Its effects were determined with the eyes shut before and after a 240 s period of conditioning with the eyes open.

Subjects wore a lightweight “helmet” that supported 3 orthogonally-aligned angular rate sensors (resolution < 0.004°/s, 100 Hz low-pass; SDG500, Systron Donner Inertial, CA). Markers on the head and helmet were digitised (Polaris Vicra, NDI, Canada) to resolve instantaneous angular velocity of the head about the GVS axis. GVS bypasses canal mechano-transduction [9, 15]. Thus, to make the real-time stimulus proportional to the canal afferent firing produced by the natural head movement, the measured signal was passed through the mechano-transduction transfer function of [10] (Fig 1A) before scaling to generate a galvanic stimulus of 0.125 mA per deg.s^-1^. Real-time data acquisition (LabVIEW Real-Time, PXI-6289 18-bit DAQ; National Instruments, TX) ensured < 1 ms point-by-point conversion and a 1 kHz output rate to the voltage controlled current stimulator (Stimsol, BIOPAC Systems, CA) so that there is effectively no delay in modulating vestibular afference. The stimulus was delivered through carbon-rubber electrodes (9 cm^2^) coated with conductive gel and fixed bilaterally over the mastoid processes. Stimulus noise was small (~0.015 mA RMS) and would have no effect on sway [22–23].

A stimulus polarity that evoked an afferent signal in the same direction as the actual head rotation was used to create a net afferent signal of the head moving faster than actual (+Sway modulation). A stimulus polarity in the opposite direction created a net signal of slower head rotation (–Sway modulation). Of course, the galvanic stimulation will not mimic vestibular function exactly as there are undoubtedly residual differences in phase relationships and linearity of gain and bandwidth, but for the purpose of this study any consistent change in afferent responsiveness to head motion allows exploration of adaptation in the balance system.

### Experiment 1. Visual recalibration of vestibular signals

In this experiment, we determined whether the CNS can adapt to a destabilizing vestibular signal (coupled or not coupled to head motion) when a reliable visual signal of body movement is available. Balance (body sway) was assessed without vision before and after a period of visual conditioning. Subjects stood on 100 mm medium-density foam with the feet together to increase reliance on the vestibular sense but without making balance unstable or difficult; resting body sway approximately doubled on inspection and subjects did not lose balance. The head faced forward and was pitched slightly upward from the primary position (Reid’s plane tilted by 18°) so that the galvanic stimulus would evoke a vestibular signal that would normally be produced by body roll [12]. To begin each trial, subjects were asked to stand still with the eyes closed for 40 s while a baseline measure of sway was obtained (Fig 1B). The movement-coupled GVS was then turned on for the next 60 s and the balance response was measured. A 240 s conditioning period followed in which subjects had the eyes open and moved the head, mostly in roll, as they made simple body movements to look around the room and throw and catch a ball, while the movement-coupled GVS remained. They then shut the eyes for 60 s for a post-conditioning test as they attempted to stand still.

The protocol consisted of four trials: (i) no galvanic stimulus (always the first trial), (ii) +Sway modulation in which the galvanic stimulus amplified the afferent response, (iii)—Sway modulation in which the galvanic stimulus attenuated the afferent response, and (iv) random modulation—a control trial in which a pre-recorded signal, independent of actual head movement, was provided in the conditioning phase, while—Sway modulation was provided in the pre- and post-conditioning phases. Between trials, subjects sat and rested for at least 5 minutes to ensure no persisting effects of the stimulation from the previous trial.

### Experiment 2. Somatosensory recalibration of vestibular signals

In a separate experiment, we determined whether the CNS can adapt to a destabilizing vestibular signal (coupled or not coupled to head motion) when a reliable somatosensory signal of body movement is available. Five subjects participated in this experiment to determine if the vestibular signal used for balance is recalibrated to reliable somatosensory afference. The experiment proceeded with the protocol of Experiment 1 using only the—Sway modulation stimulus but the conditioning period had subjects step off the foam and onto the hard floor while keeping the eyes closed. Without vision they made similar volitional body movements while keeping the feet in place. For the post-conditioning assessment they stepped back onto the foam while keeping the eyes closed.

### Experiment 3. Visual recalibration of vestibular balance reflexes

To determine how the CNS adapts to a false vestibular signal, we measured lower limb vestibular reflexes pre- and post-conditioning. Specifically, vestibular muscle reflexes were measured in 8 subjects to determine if the vestibular gain modulation and the visual conditioning altered these reflexes. A stochastic vestibular stimulus (0.25–25 Hz bandwidth, 0.54 mA RMS) was applied to evoke short- and medium-latency reflex responses in the leg muscles. This stimulus was added to the movement-coupled stimulus but was uncorrelated with the sway and statistically independent. It evokes muscular responses with the same temporal and spatial profile as those evoked by traditional square-wave GVS [24]. Reflex responses were measured during the pre- and post-conditioning phases (Fig 1B) and each of the four trials (Experiment 1) were performed twice. Electromyography (EMG) signals were recorded from surface electrodes placed bilaterally over the medial gastrocnemii (MG) and tensor fascia latae (TFL) muscles (NeuroLog, Digitimer, UK). Signals were amplified (1,000–10,000) and band-pass filtered (10–1,000 Hz).

### Measurement and analysis

The vestibular stimulus, EMG, and angular rate sensor data were sampled at 2048 Hz with 18-bit precision (PXI-6289, National Instruments, TX). Body sway was recorded at 240 Hz at the level of the C7 spinous process using a 3-D motion-tracking system (TrakSTAR, Ascension Technology Corporation, VT). We report sway amplitude as the root mean square of the position signal at C7 to provide an index of sway variability.

In Experiments 1 and 2, medio-lateral body sway (C7 level) was calculated for each test as the RMS level after low-pass filtering the raw data (10 Hz, 2^nd^ order dual-pass Butterworth).

In Experiment 3, the vestibular reflex response evoked by the stochastic stimulus was determined by calculating a cumulant density function. The inverse Fourier transform of the stimulus-EMG cross spectrum, the cumulant density function represents the time-domain relationship between the galvanic stimulus and the resulting muscle activity and typically shows the small short-latency (~60 ms) and a larger medium-latency (~100 ms) responses that are temporally and spatially similar to those obtained by trigger averaged responses to square-wave GVS [24]. EMG recordings were band-pass filtered (10–500 Hz, 4^th^ order dual-pass Butterworth) and full-wave rectified before calculating the cumulant density function using the algorithm of [25]. Data from like trials were divided to produce 30 4-second windows, providing a frequency resolution of 0.25 Hz. The cumulant density function were normalised by the product of the vector norms of the input (stimulus) and output (EMG) signals to account for differences in background EMG levels and stimulus sensitivity [22]. The peak amplitude of the larger medium-latency response (latency ~100ms) was extracted for statistical analysis. As preliminary analysis revealed no side-to-side differences in response amplitude (MG: t_39_ = 0.20, P = 0.84; TFL: t_39_ = 0.65, P = 0.52), left and right absolute response amplitudes were averaged.

Both sway amplitude (Experiments 1 and 2) and vestibular reflex responses (Experiment 3) were examined by 2-way repeated-measures analysis of variance (ANOVA). In Experiments 1 and 3, factors were conditioning (pre, post) and modulating stimulus (none, +Sway, −Sway, random). Planned comparisons of significant main effects and interactions used Dunnett’s test to compare the three galvanic stimulus trials with the no-stimulus trial, and Fisher's least significant difference (LSD) test to compare across the pre- and post-conditioning phases. In Experiment 2, factors were conditioning (pre, post) and type (control, somatosensory). A repeated-measures ANOVA of sway amplitude during the pre-stimulus phase of every trial was used to determine if baseline sway amplitude had changed. Analyses were performed using SPSS 17 (SPSS Inc., Chicago IL) with significance set at P < 0.05. Figures present mean values and 95% confidence intervals.

## Results

### Experiment 1. Visual-vestibular conditioning

**Fig 2:**
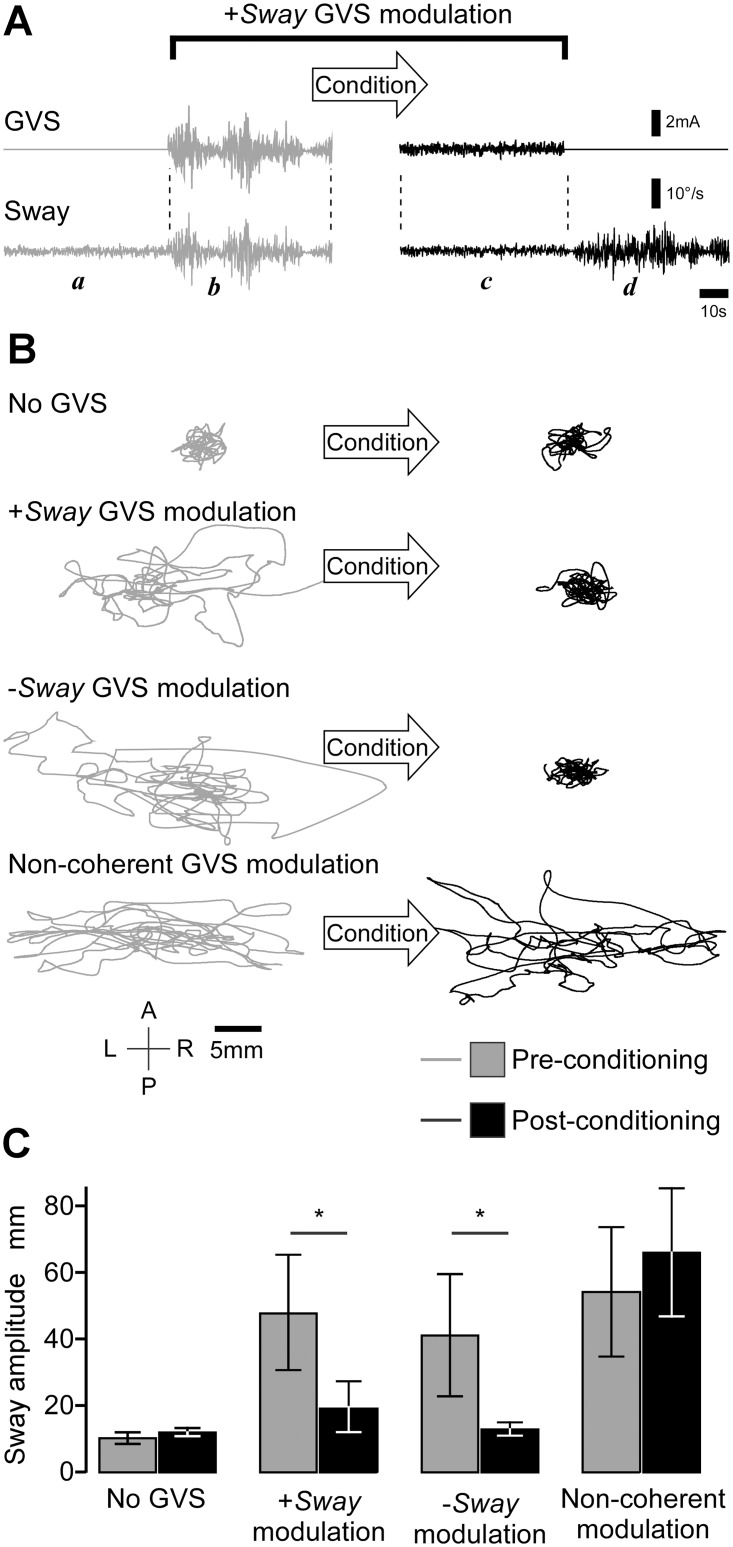
Visual-vestibular conditioning. (A) Lateral body sway and the associated galvanic stimulus from a +Sway modulated trial for a typical subject. Initially, without GVS, sway is minimal. When GVS modulated by the head movement begins, large excursions appear. After the conditioning period with the eyes open, sway returned to baseline levels despite the continuing GVS. When finally extinguished, sway increased as if the natural vestibular signal was now inappropriate. (B) Sway traces of a typical subject during the pre- and post-conditioning phases for the four tests. In the no-GVS control trial, sway was small and unaffected by conditioning. The movement-coupled galvanic stimuli (+Sway,-Sway) created a large, predominantly lateral sway but this was reversed by visual conditioning. The galvanic stimulus that was not coherent with sway produced an equivalent large increase in sway that was not reversed by visual conditioning. (C) Group mean lateral sway and 95% CI for the four trials (N = 8; * P < 0.05 by ANOVA).

Sway amplitude during the initial 40 s (no GVS) did not differ between experimental trials (F_3, 28_ = 1.88, P = 0.16). Fig 2A, which shows data of a typical subject, illustrate the essential result of the study. When the amplifying GVS was turned on there was a dramatic increase in sway (a to b) but after the conditioning period of stable balance achieved with vision available, sway returned to normal levels (c) despite the continuing GVS, only to become unstable again when the stimulus was extinguished at the end of the post-conditioning test (d).

**Fig 3:**
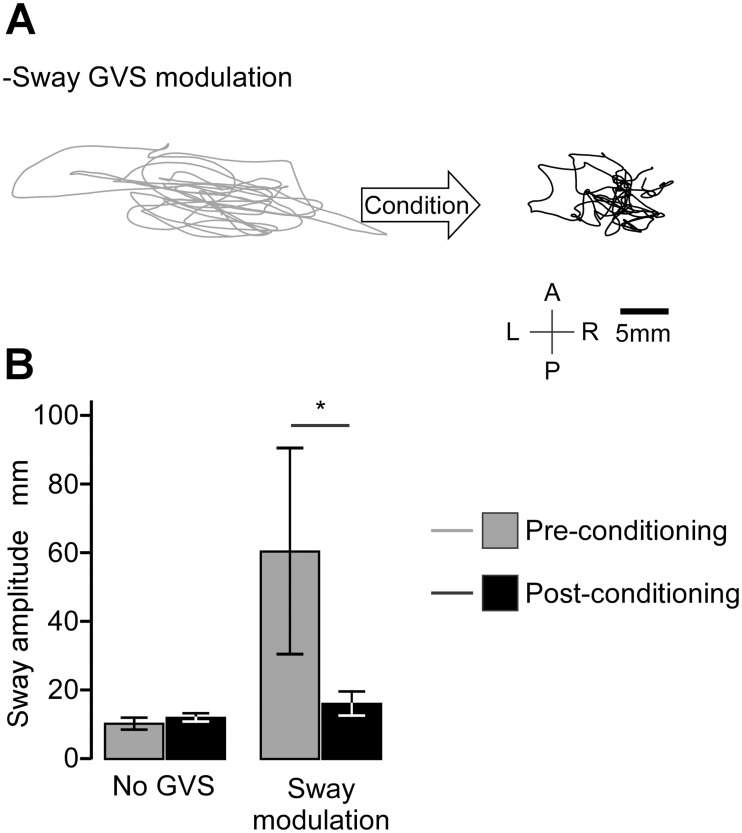
Somatosensory-vestibular conditioning. (A) Sway of a typical subject during the pre- and post-conditioning phases with the sway modulated signal. The movement-coupled galvanic stimulus created a large, predominantly lateral sway, which was reversed by the somatosensory conditioning. (B) Group mean lateral sway and 95% CI for the 4 trials (N = 8; * P < 0.05 by ANOVA).

Sway trajectories in the antero-posterior and lateral planes for all four trials are shown in Fig 3A. Compared with the control trial (No GVS), exposure to any of the galvanic stimuli resulted in large increase in sway amplitude during the pre-conditioning phases (mean 364%, SD 112%). This instability was immediately apparent to all subjects. When the galvanic stimulus was driven by the sway, either amplifying or attenuating the vestibular afferent signal, the period of visual conditioning resulted in a return to control levels of sway when tested again with the eyes closed. If the modulating vestibular stimulus was not coherent with the current sway, conditioning had no effect on sway.

Group mean results for this experiment are shown in Fig 2C. Two-way repeated measure ANOVA revealed a significant main effect of the stimulus (F_3,21_ = 14.0, P < 0.001) and a significant interaction with conditioning (F_3,21_ = 7.1, P = 0.002). In the pre-conditioning phase, sway was significantly greater (~ 4-fold) in the three stimulus trials than in the no-GVS control trial (Dunnett, P < 0.01). After the period of visual conditioning, sway amplitude was significantly reduced for both the +Sway and—Sway modulation stimuli (LSD, P < 0.05 for each) and not different to no-GVS control levels (Dunnett, P = 0.12). When the vestibular stimulus had the same intensity and bandwidth but was uncoupled from current head motion, visual conditioning had no effect on sway (LSD, P = 0.56), which remained significantly greater than no-GVS control levels (Dunett, P < 0.001).

### Experiment 2: Somatosensory-vestibular conditioning

Fig 3 shows the results of Experiment 2 for a typical subject (3A) and for the group (3B). As in Experiment 1, sway amplitude increased ~4-fold with sway-coupled GVS during the preconditioning tests (LSD, P = 0.003). Subjects then stepped off the foam onto hard floor to condition body and head movements with the eyes closed. This somatic-vestibular conditioning significantly reduced sway amplitude (LSD, P = 0.025) so that it was not different from control trials (LSD, P = 0.21).

### Experiment 3: Visual-vestibular conditioning effects on vestibular reflexes

**Fig 4:**
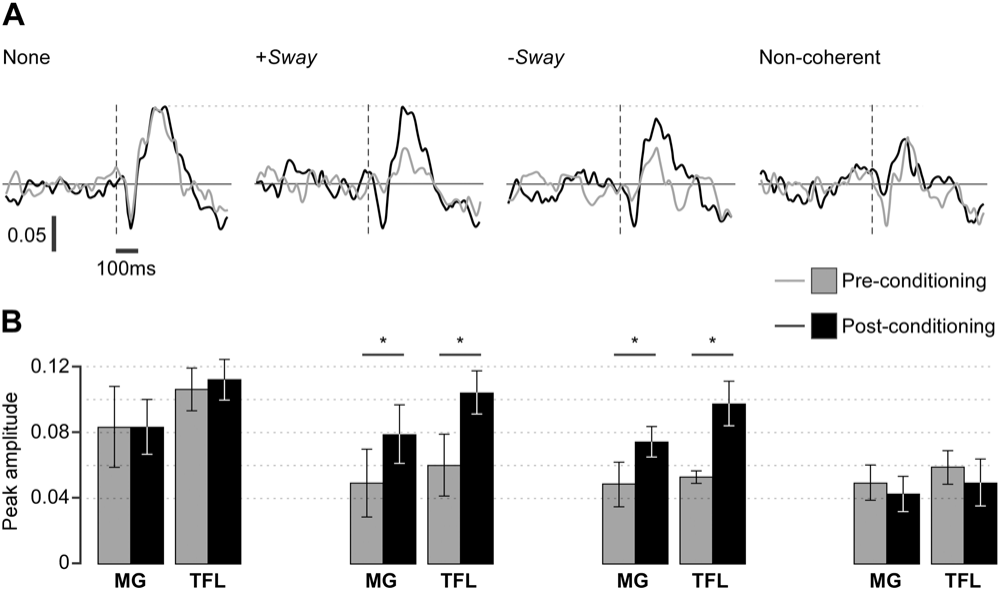
Vestibular reflex responses to galvanic stimuli. (A) For a typical subject, responses in right tensor fascia lata muscle (TFL) muscle measured as cumulant density to stochastic galvanic stimulation are shown for each GVS modulation stimulus, before (gray) and after (black) visual conditioning. The biphasic short-latency (~50ms) and long-latency responses are evident. (B) Group mean of peak medium latency response amplitude with 95% CIs in medial gastrocnemius (MG) and TFL. * P < 0.05.

Typical EMG responses evoked by the stochastic vestibular stimulus in the right TFL muscle are shown in Fig 4A. This stochastic stimulus used to identify the response in vestibular reflex pathways is unrelated to the sway modulated stimuli being examined. Without a sway-modulated stimulus, robust biphasic reflex responses were seen with a short-latency response at ~50 ms and a medium-latency response at ~100 ms. Similar responses were observed in both muscles (TFL, MG) bilaterally. Both the sway-modulated stimulus and the non-coherent stimulus markedly reduced the short- and medium-latency vestibular reflex responses compared with the no-stimulus trials (compare gray plots). After visual conditioning of the sway-modulated stimuli, the responses increased in amplitude to approach the levels of the no-stimulus control (black lines). This was not seen with the visual conditioning of the non-coherent stimulus.

**Fig 5:**
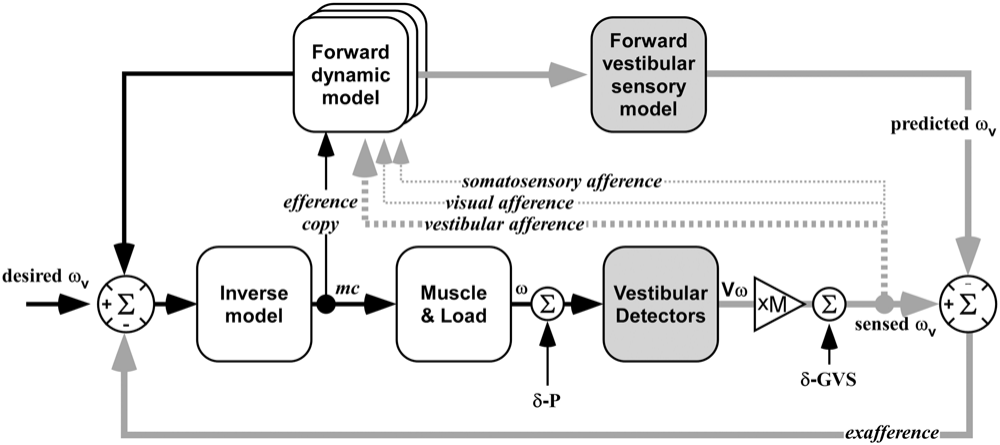
Model of vestibular sensory control for balance. Motion sensed by the vestibular system (gray arrows: sensed ω_v_) is aligned with desired motion (desired ω_v_) by a forward prediction of the vestibular response to the command (predicted ω_v_) to identify disturbances (exafference signal). Disturbances are corrected by passing exafference through an inverse model of the motor response (motor command mc to sensed ω_v_). We change vestibular afferent gain at xM. Motor reflex responses are probed with an independent perturbation (δ-GVS). Initially, a gain change creates an exafferent signal that increases sway as it is not aligned with predicted ω_v_. When conditioned with another sensory channel, the forward model changes the exafference to reafference, which is cancelled. The perturbation, δ-GVS, evokes the same balance response in the calibrated system.

Group mean amplitudes of the medium latency response for both TFL and MG show the same pattern (Fig 5B), with statistical significance seen in the modulating stimulus (F_3,2_ = 10.6 [TFL] and 36.4 [MG], P < 0.001) and stimulus-conditioning interaction (F_1,4_ = 8.2 (TFL] and 14.2 [MG], P < 0.003). Pre-conditioning vestibular-evoked muscular responses were approximately halved in both muscles compared with the no-stimulus control (Dunnett, P < 0.005). After visual conditioning, there was a significant increase in the amplitude of vestibular-evoked muscular responses for the sway-modulated stimuli in both muscles (LSD, P < 0.005) so that they were no longer different from those of the control trial (Dunnett, P = 0.26). This was not so for visual conditioning of the non-coherent stimulus where there reflex muscle responses were unchanged by conditioning (LSD, P = 0.38) and remained different from the control (Dunnett, P < 0.005).

## Discussion

A rapid change in vestibular sensitivity disrupts balance until a period of stable balance is achieved by other sensory inflow. When stable balance is achieved, the vestibular signal becomes useful for balance control rather than a disturbance. A vestibular signal with no relationship with head motion cannot be transformed in this way nor overridden, at least in the short term, so that it continues to create a strong disturbance. Clinically, we see this in pathological situations where aberrant vestibular discharge has dramatic effects on balance compared with the situation of compensated loss of canal afference.

### Afferents and pathways

GVS evokes a strong afferent signal of head rotation [12–13, 16, 18, 26]. Thus, the adaptation observed here must include an action on signals of semicircular canal origin. Although an otolithic balance response to GVS has been more difficult to identify [16, 20–21, 27–29], otolithic afferent stimulation undoubtedly evokes some net signal of linear acceleration [11], which, if significant, must also be subject to these adaptive processes. The present study provides no evidence for the descending pathways recruited but it would not be surprising if it included more than one of the vestibulospinal, reticulospinal or corticospinal tracts.

### Feedback control?

Pathological loss of vestibular sensor gain is associated with increased sway [30–31], suggesting that increasing sensor gain by +Sway modulation might reduce sway. However, high gain coupled with long conduction delays creates instability in reflex-feedback loops [32]. If the increased sway observed with +Sway modulation resulted from instability in the pathways of balance control, we would expect reduced sway with-Sway modulation. Instead we see similar increases in sway with both modulations (Fig 2B). This leads us to conclude that the vestibular influence on balance is not through a simple negative feedback loop, which is in agreement with the very low loop gain (approximately unity) estimated for balance control pathways [33].

### Sensory reweighting?

When the non-coherent vestibular stimulus was first delivered without visual cues it evoked a balance-correcting response that increased sway. During conditioning, visual and somatosensory signals of body sway allowed successful balance. The sensory reweighing hypothesis suggests that adaptation would up-weight visual and somatosensory cues and down-weight vestibular cues [5]. This is what appears to occur when the CNS is repeatedly exposed, over the period of days to months, to non-coherent vestibular stimulus [2–3]. However, this was not observed in the present study. Visual conditioning did not reduce sway with the non-coherent vestibular signals (Fig 2) and did not increase vestibular-evoked muscle reflexes (Fig 4).

Unlike visual disturbances, self-triggered, anticipated and unexpected vestibular stimuli all evoke identical responses that do not extinguish with repetition within a single session [34]. As visual and somatic signals can represent self- or surround-motion, there must exist flexible weighting networks to deliver appropriate balance responses for different settings. In contrast, a physiological vestibular signal can only represent self-motion in the gravito-inertial frame of balance control so there is not the same biological need to suppress vestibular responses. With acute exposure to non-coherent vestibular stimulus, the lack of suppression of vestibular responses is consistent with the greater weight placed on unreliable vestibular cues over visual cues in perceiving self-motion [35].

We conclude, cautiously, that sensory reweighting for balance control does not operate on a vestibular error signal (exafference) in the minute-to-minute adaption for balance control. This argument does not apply to the longer-term processes of accommodation and compensation [2–3, 36] typical after vestibular injury or chronic exposure to non-coherent vestibular stimuli.

### Reafference and prediction

The results are considered in terms of the related models of reafference [37] and forward sensory prediction [38]. This model (Fig 5) identifies causality between motor output and sensory inflow to predict the sensory outcomes of motor actions and allow vestibular reafference (sensory consequence of action) and exafference (sensory consequence external forces) to be extracted from the vestibular signal [39–40].

Before conditioning, both the +Sway and-Sway modulation created an afferent error signal, or exafference, that was treated as an external perturbation. During conditioning this afference acquired new meaning as the reafferent consequence of motor action making it useful for monitoring and correcting motor actions, and for creating the forward model for correcting external disturbances. The CNS found a solution that generated the same behavioural outcome so that sway returned to normal.

This solution was not found for non-coherent vestibular afference, which remained as exafference so that the uncontrolled sway was unchanged after conditioning. Thus, correlation with an efferent or efferent-copy signal is critical for the CNS to identify the reafferent component of total vestibular sensory inflow so that it only responds to the exafferent component of the signal. We see in these results that the forward sensory model persists for long periods until a period of stable balance is achieved through other sensory inflow.

The reflexes evoked by the stochastic stimulus (Fig 4) give clues about the control process. First exposure to the sway-modulated stimulus halved their pre-stimulus level. This is explained by the vestibular reflex being swamped by larger balance responses that could arise from direct vestibular action or volitional reactions based on perception [41–42]. Conditioning restored the reflex as the balance system treated the modulated vestibular afference (+Sway and-Sway) as reafference of its motor output rather than an external disturbance. A recalibrated vestibular forward sensory model [8] can now predict and cancel this reafference to estimate the stochastic stimulus disturbance to drive an appropriate reflex output. This contradicts the reweighting hypothesis prediction of reduced vestibular reflexes if conditioning determines that they were not useful for the task.

When +Sway modulation amplified the afferent signal of head movement, recalibration at the level of vestibular afference would mean the stimulus that elicited the reflex represented a smaller real disturbance. This recalibration would evoke a smaller post-conditioning reflex if the rest of the control network remained unchanged. However, conditioning to the amplified vestibular signal aligns the desired, predicted and sensed movement (ω) as if there were no external postural disturbances (δ-P). Recalibration, by updating the forward model, changes the exafference of the modulated signal into reafference that is cancelled from total vestibular afference (summation of the vestibular reafference and exafference). The residual vestibular exafference (probed by stochastic GVS) is not recalibrated so that δ-P always evokes the same balance response in a calibrated system.

There is strong neurophysiological evidence for this vestibular processing. At early stages of processing, vestibular nuclei neurons distinguish the reafference of active motion from the exafference of passive motion [39] while neurons in the cerebellar rostral fastigial nuclei code selectively for exafference [43]. For this vestibular processing to cancel reafference and extract exafference, Brooks and Cullen [44] show that there must be no discrepancy between the predicted and actual proprioceptive sensory consequences of self-motion. This neural mechanism could contribute to the recalibration of the GVS-modulated gain change of vestibular afference described here. In Experiment 2, vestibular processing cannot discriminate active from passive self-motion without a reliable somatosensory signal. We propose that reliable visual afference serves the same role.

## Conclusions

An erroneous vestibular signal has profound effects on balance. If it is unrelated to head motion, the CNS has no immediate mechanism of reweighting it to reduce its influence on balance. If it is causally related to head motion, the CNS will reinterpret and use it to restore balance stability. Recalibration of a forward sensory control model explains this. These adaptations occur during a short period when stable balance is achieved, which in these studies was through another reliable sensory cue.
